# Association Between Oral Dysbiosis and Alzheimer’s Disease: A Systematic Review

**DOI:** 10.3390/jcm14103415

**Published:** 2025-05-13

**Authors:** Valeria Martínez-Martínez, Francisco Javier Rodríguez-Lozano, María Pilar Pecci-Lloret, Nuria Pérez-Guzmán

**Affiliations:** Dermatology, Stomatology, Radiology and Physical Medicine, Hospital Morales Meseguer, Medicine School, University of Murcia, 30100 Murcia, Spain; valeria.m.m@um.es (V.M.-M.); mariapilar.pecci@um.es (M.P.P.-L.); nuriap_g@hotmail.com (N.P.-G.)

**Keywords:** periodontal microorganisms, periodontal pathogens, oral health, oral dysbiosis, oral disease, Alzheimer’s disease

## Abstract

**Objective**: The main objective of this systematic review is to select and critically synthesize the available evidence from studies that aimed to verify whether there is a relationship between dysbiosis of the oral cavity and the development of Alzheimer’s disease. **Methodology**: A search was conducted on 30 November 2024 and updated on 9 January 2025, in the PubMed, SciELO Scopus, and Web of Science databases, limiting the search to the last 5 years. The review was carried out under the criteria of the PRISMA 2020 guide for systematic reviews and has been accepted into the PROSPERO registry (CRD42025636275). We analyzed the risk of bias of studies using the JBI guidelines. **Results**: Initially, 2009 articles were obtained. After eliminating duplicates, we obtained 1716; of these, following the inclusion and exclusion criteria, 185 articles were reviewed by title and abstract, discarding 171. Of the remaining 14 articles, 12 final articles were selected. In the results obtained, it has been observed that there is a relationship between inflammation derived from oral dysbiosis caused by periodontal disease and its extension to the neuronal tissue via the hematogenous blood–brain barrier (BBB) and nerve (V pair). Among the most frequently found oral microbiota are *Veillonella*, *Fusobacteria*, *Prevotella*, *Porphyromonas*, *Lactobacillus*, and *Streptococcus*. **Conclusions**: Oral dysbiosis gives rise to the establishment of inflammatory processes that lead to neurological degeneration, either through its passage across the blood–brain barrier or by a direct connection between the free nerve endings of the periodontium and the proprioceptors found in the central nervous system. Therefore, the chronic inflammation caused by oral dysbiosis and its role in systemic inflammation could be associated with the onset and progression of Alzheimer’s disease (AD); however, more studies are needed to show the association between oral dysbiosis and Alzheimer’s disease.

## 1. Introduction

Currently, it is well known that the human body is colonized by a vast number of microorganisms—between 10 and 100 trillion—including bacteria, viruses, fungi, and archaea [1]. The oral cavity harbors a heterogeneous and complex microbial community, resulting in a unique ecosystem known as the oral microbiome [2,3]. Among the bacterial microorganisms, six main phyla have been identified: Actinobacteria, Bacteroidetes, Firmicutes, Proteobacteria, Spirochetes, and Fusobacteria [2]. Although the majority of these microorganisms are classified as non-pathogenic, a small subset exhibits opportunistic pathogenic characteristics [2,3]. When alterations occur in this microbial balance, the emergence of these opportunistic pathogens is favored, leading to dysbiosis within the ecosystem [4].

This imbalance is currently on the rise, as a relationship has been established between systemic diseases and dysbiosis—both intestinal and oral—revealing that these diseases may occur as a result of bacterial imbalance associated with multiple factors [4,5]. The oral microbiome is the second most prevalent microbial ecosystem in the human body, following the gut microbiome, and includes over 700 bacterial species [2,6].

In addition to its diversity, the oral microbiota plays a key role in physiological and immunological functions. These include the biotransformation and elimination of environmental chemical compounds, modulation of immune responses, regulation of homeostasis between pro-inflammatory and anti-inflammatory processes, as well as the inhibition of adhesion, proliferation, and invasion of pathogenic microorganisms [6,7]. Accumulating evidence suggests a possible connection between neurodegenerative disorders and the so-called “oral–brain axis”, resulting in numerous clinical studies examining the association between the oral microbiome and Alzheimer’s disease (AD) [6,8]. The potential role of this axis in the pathogenesis of AD lies in the ability of periodontal pathogens and/or their toxins to trigger diseases related to AD, inducing pathological changes and cognitive impairment [9].

The literature describes that both the onset and progression of AD are linked to microbial dysbiosis of the intestinal and oral cavities. This dysbiosis can initiate and accelerate the formation of neurofibrillary tangles and β-amyloid plaques [10].

Despite the growing number of studies, the evidence regarding whether a real connection exists between the oral microbiome and AD remains unclear, and it is still considered a hypothesis yet to be proven [11]. Nonetheless, the study of the oral microbiota offers numerous advantages, among which we highlight the following:

The oral microbiome exhibits significant individual variability and shows differences depending on the various anatomical niches within the oral cavity [11]. Its alteration due to various factors has been linked to susceptibility and development of major systemic diseases such as cardiovascular diseases, osteoporosis, diabetes, obesity, metabolic disease, pregnancy complications, respiratory diseases, rheumatoid arthritis, chronic kidney disease, and AD [12,13,14].

Therefore, the importance of studying the oral microbiome lies in the connection between pathogenic oral microorganisms that cause inflammation in the oral cavity and neurodegeneration and cognitive decline, highlighting its high potential for study in our field [15].

Thus, the main objective of this systematic review is to select and critically synthesize the available evidence of studies aiming to determine whether there is an association between oral cavity dysbiosis and the development of AD.

## 2. Materials and Methods

This systematic review was conducted in accordance with the PRISMA 2020 guidelines, an acronym for “Preferred Reporting Items for Systematic Reviews and Meta-Analyses” [16]. It has been accepted in the PROSPERO registry (CRD42025636275).

### 2.1. Article Selection: Inclusion and Exclusion Criteria

For this systematic review, we selected articles that investigated the potential influence of oral dysbiosis on the onset, development, progression, and worsening of AD. Articles were included if they were published within the last five years, written in English or Spanish, involved human subjects, and aimed to explore the connection between oral microbiome alteration—such as that seen in periodontal disease—and the onset of AD.

On the other hand, we excluded articles published before 2020, studies involving animal samples, and research analyzing samples that were not part of the oral microbiome or the oral cavity itself—such as studies focused on intestinal or fecal microbiota.

We also excluded articles involving other neurodegenerative diseases, such as Parkinson’s disease, as well as studies that linked other variables (in addition to oral dysbiosis) with AD (e.g., cardiovascular disease, diabetes). Thus, any article not meeting the inclusion criteria described above was excluded.

The inclusion criteria followed the PECO model (Population; Exposure Comparison/Control; Outcome):

- Population: Patients with Alzheimer’s disease.
- Exposure: Altered oral microbiota (dysbiosis);
- Comparison/Control: Healthy individuals.
- Outcome: Relationship between altered oral dysbiosis and Alzheimer’s disease in patients.

Research Question: Is there an association between oral dysbiosis and Alzheimer’s disease?

### 2.2. Search Strategy

#### 2.2.1. Information Sources

An exhaustive electronic search was conducted across the databases PubMed (Medline), SciELO, Scopus, and Web of Science. The search was limited from the beginning to the last five years in order to review the most updated information. It was initiated in November 2024, continued throughout December, updated in January 2025, and finalized in April 2025.

The process of searching, selecting, data extraction, and quality analysis was carried out by researchers VMM, FJRL, and MPPL.

#### 2.2.2. Search Terms

The search was performed using the following keywords: “periodontal microorganisms”, “periodontal pathogens”, “oral health”, “oral dysbiosis”, “oral disease”, “Alzheimer”. Boolean operators AND and OR were used.

The common search formula used across all databases (Medline, SciELO, Scopus, Web of Science) was:

(((((periodontal microorganisms) OR (periodontal pathogens)) OR (oral health)) OR (oral dysbiosis)) OR (oral disease)) AND (alzheimer).

#### 2.2.3. Study Selection

After performing the search, the results were imported into the bibliographic manager Zotero to enable a more precise and rigorous article selection. The reference manager allowed us to remove duplicates and organize the articles into categorized collections.

Initially, after removing duplicates, articles were screened by title and abstract according to the inclusion and exclusion criteria mentioned above. Then, full texts were read and thoroughly analyzed for inclusion in the results section of this systematic review.

#### 2.2.4. Quality Assessment

To evaluate the quality of the case-controls selected for this systematic review, the Joanna Briggs Institute Critical Appraisal tools for use in Systematic Reviews Checklist for Case Control Studies were used. Specifically, the checklist for cohort, case-control, and cross-sectional studies was applied.

The checklist consists of 10 items that assess the quality of information across different sections of the articles:

1. Were the groups comparable other than the presence of disease in cases or the absence of disease in controls?
2. Were cases and controls matched appropriately?
3. Were the same criteria used for identification of cases and controls?
4. Was exposure measured in a standard, valid and reliable way?
5. Was exposure measured in the same way for cases and controls?
6. Were confounding factors identified?
7. Were strategies to deal with confounding factors stated?
8. Were outcomes assessed in a standard, valid and reliable way for cases and controls?
9. Was the exposure period of interest long enough to be meaningful?
10. Was appropriate statistical analysis used?

There is no predefined scale to classify high, moderate, or low risk using this checklist. Instead, authors must decide based on which items are rated as “No” or “Unclear”. For this reason, we considered this aspect in our evaluation, in addition to using the following classification as a guiding reference.

- Low risk: 9–10 items
- Moderate risk: 6–8 items
- High risk: 0–5 items

Each checklist item was rated as “Y” if the article met the criterion, or “N” if it did not, or “U” if it was unclear.

On the other hand, to determine the risk of bias of the selected cross-sectional studies, the Joanna Briggs Institute Critical Appraisal tools for use in Systematic Reviews—checklist for analytical cross-sectional studies were used.

The checklist consists of 8 items that assess the quality of information across different sections of the articles:

1. Were the criteria for inclusion in the sample clearly defined?
2. Were the study subjects and the setting described in detail?
3. Was the exposure measured in a valid and reliable way?
4. Were objective, standard criteria used for measurement of the condition?
5. Were confounding factors identified?
6. Were strategies to deal with confounding factors stated?
7. Were the outcomes measured in a valid and reliable way?
8. Was appropriate statistical analysis used?

As in the other checklist, there is no predefined scale to classify high, moderate, or low risk using this checklist. Instead, authors must decide based on which items are rated as “No” or “Unclear”. For this reason, we considered this aspect in our evaluation, in addition to using the following classification as a guiding reference.

- 1–4 High
- 5–6 Moderate
- 7–8 Low

#### 2.2.5. Data Extraction

Data extraction from the selected articles was performed by classifying them into the following categories: author, year of publication, study type, sample type, sample collection method, and whether or not an association between oral dysbiosis and AD was found.

## 3. Results

### 3.1. Article Selection and Flow Diagram

Following a thorough search across the PubMed, SciELO, Scopus, and Web of Science databases, a total of 2009 references were retrieved (1223 from PubMed, 30 from SciELO, 517 from Scopus, and 239 from Web of Science). These references were imported into the Zotero reference manager, where 293 duplicate studies were removed, resulting in 1716 articles screened by title and abstract.

Of these, 1692 studies were excluded for not meeting the established eligibility criteria: studies published within the last five years, full-text availability in English or Spanish, and studies not classified as cross-sectional, cohort, randomized clinical trials, or case-control studies. Consequently, 14 articles were selected for full-text analysis.

**Figure 1:**
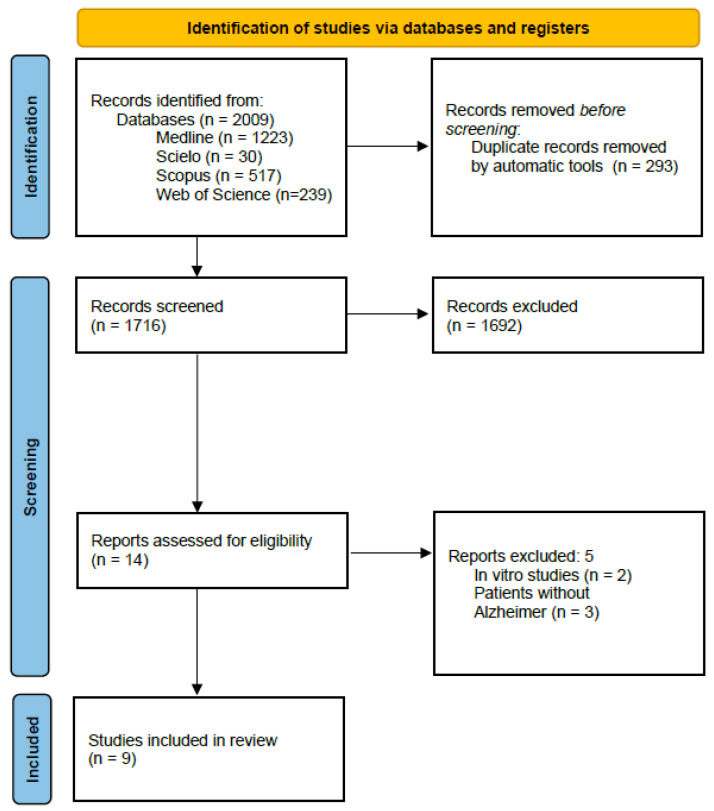
Search flow diagram of article selection from PubMed, SciELO, Scopus, and Web of Science databases.

Subsequently, two studies were excluded for being in vitro studies, and three others were excluded for not meeting the PECO model criteria (P = Patients with Alzheimer’s disease). Finally, nine articles were selected and included for analysis in this systematic review (Figure 1).

### 3.2. Quality Assessment Results of the Included Studies

A quality assessment of the selected studies was conducted, as presented in Table 1 and Table 2, which outlines the overall risk of bias found in the articles. This table was developed based on the JBI Critical Appraisal Checklist [17], one specifically designed for case-control and another for cross-sectional studies, which are the types included in this systematic review. Following the evaluation, it was determined that the risk of bias in all cases and controls is low and moderate, with scores ranging from 6 to 9. On the other hand, the cross-sectional study’s risk of bias was low, with a score of 8.

It should be emphasized that all the articles received an ‘unclear’ response for item 9, which refers to whether the period of exposure was sufficiently long, since, as stated in their limitations, it cannot be determined whether the changes in the microbiota precede or follow the disease. On the other hand, all the articles received a “yes” response for items 1, 8, and 10, which refer to “Were the groups comparable, other than the presence of disease?”, “Were the outcomes measured in a valid and reliable way?”, and “Was appropriate statistical analysis used?”, respectively. In summary, three articles present a low risk of bias, while six articles present a moderate risk of bias.

### 3.3. Data Extraction Results

After the final selection of articles, data were analyzed and extracted according to the parameters described in Section 2.2.4. The data collected are presented in Table 3. As previously mentioned, all reviewed articles were published within the last five years to ensure the most up-to-date information. Each of them examines the potential relationship between AD and oral dysbiosis. Furthermore, all studies meet the criteria of being cross-sectional or case-control studies and were assessed using the STROBE guidelines [26]. It is important to note that all included studies were conducted in human subjects.

**Figure 2:**
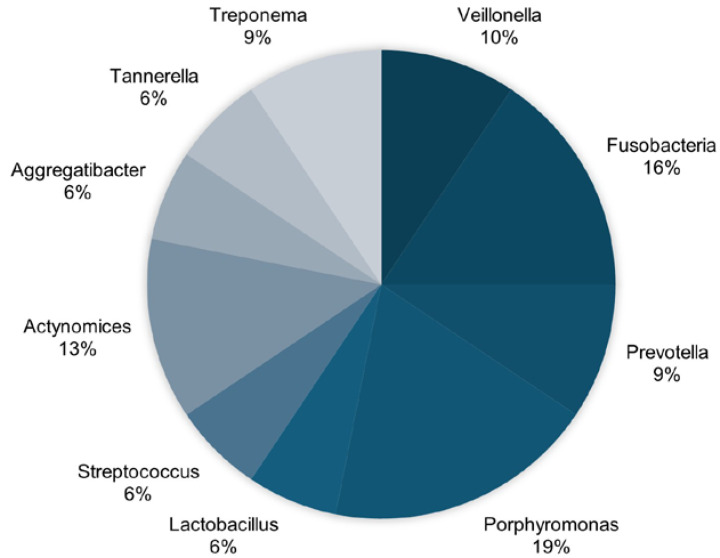
Most prevalent oral microbiota in AD.

Regarding the oral microbiome analyzed, the most frequently identified bacteria in the studies include Porphyromonas, Fusobacteria, Veillonella, and Actinomyces. Oral dysbiosis caused by these bacteria—as well as others such as Prevotella, Treponema, Streptococcus, Aggregatibacter, Tannerella, and Lactobacillus—is associated with the onset of degenerative neuroinflammatory processes that lead to the development of AD (Figure 2).

### 3.4. Bibliometric Analysis

**Figure 3:**
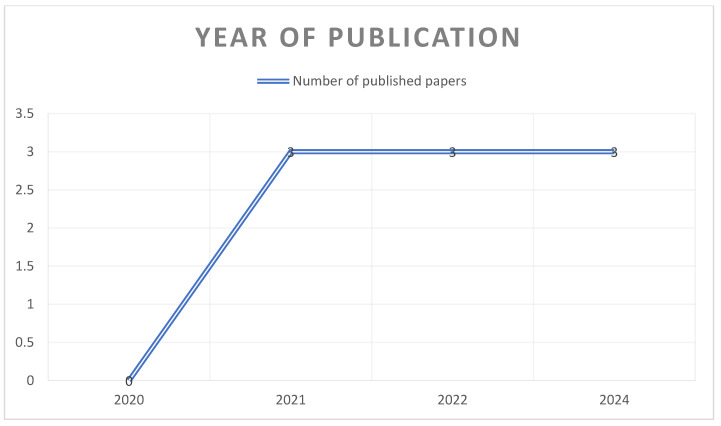
Year of publication of the studies.

**Figure 4:**
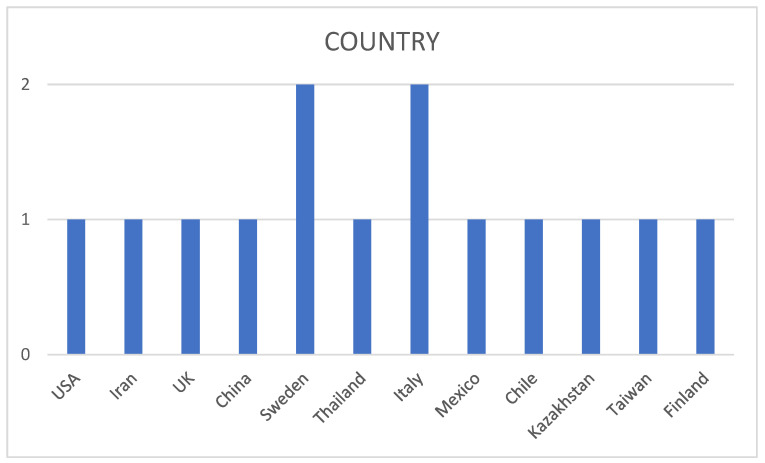
Number of studies published by country.

**Figure 5:**
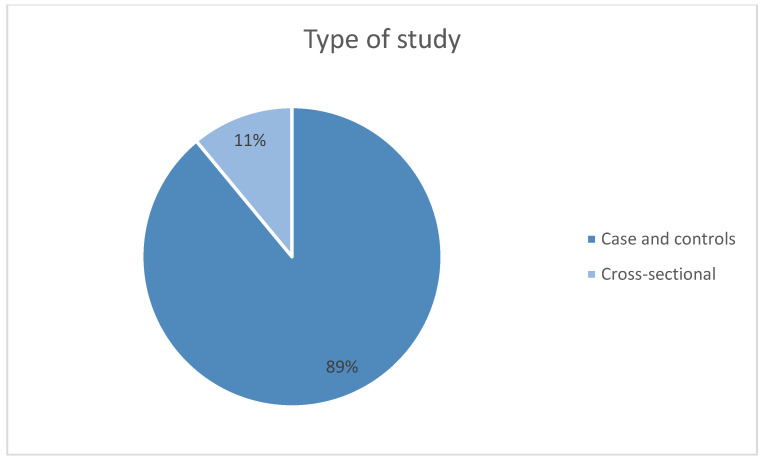
Number of studies published by type of study.

In the present review, the articles have been categorized according to the year of publication (Figure 3), the country of publication (Figure 4), and the type of study (case-control studies, cross-sectional studies) (Figure 5).

As previously mentioned, regarding the year of publication, all the articles were published within the last five years. First, three of the reviewed articles were published in 2021; subsequently, another three articles were published in 2022 and 2024, respectively, on the topic under discussion (Figure 3).

Due to the expected increase in the incidence of AD over the coming decades, as well as the publication of studies such as those reviewed in this work demonstrating the existing connection between oral dysbiosis and this disease, an increase in publications on this topic is anticipated in the coming years.

Regarding the countries of publication, most of the analyzed articles were published by Sweden and Italy. The remaining countries each contributed a single publication (Figure 4).

Similarly, the analyzed studies have been classified as case-control studies and cross-sectional studies, with the majority being cross-sectional. A total of eight articles (89%) belong to the case-control study group, while one article (11%) corresponds to cross-sectional studies (Figure 5).

## 4. Discussion

The link established between periodontal disease and the intensification of systemic inflammation translates into a decline in cognitive function and progression of neuronal degeneration. There are various pathways through which bacterial microorganisms access the neural system, leading to Aβ (alpha-beta) deposition in brain tissue. Among these are intestinal microbiome dysbiosis, damage to the oral mucosa, and thereby hematogenous transmission of pathogens to the blood–brain barrier (BBB) as a consequence of local inflammatory processes that spread and reach the central nervous system. Therefore, among the risk factors for the worsening of AD, we find changes in the microbiota, particularly the oral microbiota [20,28].

Recent experimental studies suggest that systemic inflammation plays a fundamental role in the onset of both dementia and periodontal disease. Likewise, an increase in inflammatory markers associated with periodontal pathogens has been observed in people with AD. Thus, the evidence supporting this link between both diseases can serve as a basis for promoting oral health initiatives aimed at preventing dementia [29,30]. Moreover, lipopolysaccharides (LPS) from periodontal bacteria have been detected in the brains of patients with AD, suggesting a direct microbial insult that may exacerbate amyloid-β accumulation and tau hyperphosphorylation—hallmarks of Alzheimer’s pathology. This neuroinflammatory milieu disrupts neuronal homeostasis, promoting synaptic dysfunction, neuronal loss, and cognitive decline [29,30].

In chronic periodontitis, responsibility is attributed to one of the most important pathogens causing the disease, Porphyromonas gingivalis (Pg) [3]. Numerous studies confirm that the immune response is altered by this bacterium, which invades epithelial and endothelial cells, stimulates cell proliferation, and favors tumor cell migration due to its inhibition of p53 (a tumor suppressor). Moreover, it disrupts oral microbiota homeostasis, promoting the pathogenic nature of the bacterial oral biofilm. After altering the host’s immune response and its interaction with the bacterial biofilm, it triggers a local inflammatory reaction that can lead to a chronic low-grade systemic inflammatory state [27,31]. This is why Pg is considered a pathogen that may act as a cofactor in the development of neurodegenerative diseases such as AD, due to its ability to access the bloodstream or reach the brain via neural pathways, as a result of inflammatory mediators and transient bacteremia [27].

In most of the studies analyzed in this review, an association between Pg and AD was found. Franciotti et al. confirmed the existence of a relationship between the abundance of Pg in the oral cavity and neurodegenerative diseases [27]; likewise, Wu et al. supported the findings of this bacterium in patients with AD, establishing that by inducing a chronic inflammatory state in an area close to the central nervous system, such as the oral cavity, its penetration into the BBB and invasion of brain tissue were more likely, implicating it in the neuroinflammation characteristic of the disease under study [24]. Taati Moghadam et al. [20], referencing the sequencing of the Porphyromonas genus genome in the oral cavity, reported an increase in these bacteria in patients with AD compared to controls.

Other authors, such as Sansores-España et al. [22], quantified the presence of P. gingivalis in deep periodontal pockets, finding that periodontal inflammation and cognitive status are interrelated due to the significant quantity of Pg found in patients with AD. In fact, they established an association between proinflammatory cytokines and certain levels of Pg in periodontal pockets. However, although the hypothesis regarding the role of this bacterium in AD is gaining strength, some authors have not obtained significant results concerning its prevalence in the topic under discussion [19,23]. Nevertheless, despite the lack of correlation, Kamer et al. [32] found that Porphyromonas catoniae was elevated in the P-Tau group, although it was inconsistent in the histogram, thus questioning its association with AD. Similarly, Panzarella et al. [21] did not show significant differences in antibodies against Treponema denticola and Porphyromonas gingivalis in the cerebrospinal fluid and serum of subjects with AD.

On the other hand, Sritana and Phungpinij [18] established that certain levels of oral microbiota, specifically from the families Peptostreptococcaceae and Fusobacteriaceae, were increased in patients with AD. Similarly, Panzarella et al. [21] found in their study that patients with poor oral health associated with chronic periodontitis suffered from AD, reporting a significant increase in the species Fusobacterium nucleatum from the Fusobacteriaceae family. Subgingival bacterial dysbiosis and its association with the β-amyloid-positive patient group in the study by Kamer et al. [32] did reveal enrichment in Porphyromonas endodontalis and Treponema parvum, although a higher richness of species such as Fretibacterium, Prevotella, and Dialister was also noted.

Conversely, studies like that of Wu et al. [24] explain that bacteria from the genera Lactobacillales, Streptococcaceae, and Firmicutes/Bacteroidetes are increased in AD. Others found particularly abundant taxonomic units such as Slackia exigua, which were also associated with deep periodontal pockets, and Lachnospiraceae [19].

The main limitation of this systematic review is the limited number of published articles on the impact of oral microbiota on AD conducted in human patients, as many of the results found were based on in vitro samples or animal studies. Additionally, many studies focused on the gut microbiota.

Based on the results obtained in this work, the role of oral dysbiosis in AD appears consistent, both in its etiopathogenesis and progression. This is due to the abundance of various oral pathogenic microorganisms that can negatively impact oral health, microbiomes, and the body. There is a clear need to continue investigating the oral microbiota as a potential non-invasive biomarker aimed at predicting patients’ risk of developing AD or the progression of cognitive decline once the disease is established.

## 5. Conclusions

Taking into account the aforementioned limitations, it can be concluded that all the included studies support the existence of an association between both conditions, determining that one of the possible pathways for the onset and progression of Alzheimer’s Disease (AD) could be the disruption of the oral microbiome balance as a result of chronic inflammatory processes derived from periodontal disease. In a state of dysbiosis, the main pathogenic microorganisms colonizing the oral microbiome are P. gingivalis, T. forsythia, and T. denticola, all of which are associated with the development of Alzheimer’s Disease. Further studies are needed to confirm these hypotheses.

## Figures and Tables

**Table 1:** Quality assessment of case-control studies; JBI Critical Appraisal Checklist [17].

Key Items	Sritana, N. et al. (2024) [18]	Holmer et al. (2021) [19]	Taati Moghadam et al. (2022) [20]	Panzarella et al. (2022) [21]	Sonsores-España et al. (2022) [22]	Issilbayeva et al. (2024) [23]	Wu et al. (2021) [24]
**1**	Y	Y	Y	Y	Y	Y	Y
**2**	N	Y	N	N	N	Y	N
**3**	Y	Y	Y	Y	Y	Y	Y
**4**	Y	Y	Y	Y	Y	Y	Y
**5**	Y	Y	Y	Y	Y	Y	Y
**6**	N	Y	Y	Y	N	Y	N
**7**	N	Y	N	Y	N	N	N
**8**	Y	Y	Y	Y	Y	Y	Y
**9**	U	U	U	U	U	U	U
**10**	Y	Y	Y	Y	Y	Y	Y
**Total risk of bias**	**6 Yes** **3 No** **1 Unclear** **Moderate**	**9 Yes** **1 Unclear Low**	**7 Yes** **2 No** **1 Unclear** **Moderate**	**8 Yes** **1 No** **1 Unclear** **Low**	**6 Yes** **3 No** **1 Unclear Moderate**	**8 Yes** **1 No** **1 Unclear** **Low**	**6 Yes** **3 No** **1 Unclear** **Moderate**

**Table 2:** Quality assessment of cross-sectional studies; JBI Critical Appraisal Checklist.

Key Item	Qiu et al. (2024) [25]
**1**	Y
**2**	Y
**3**	Y
**4**	Y
**5**	Y
**6**	N
**7**	Y
**8**	Y
**Overall**	**7 Yes** **1 No** **Low**

**Table 3:** Extracted data of the selected articles.

**Author**	**Study Type**	**Sample and Group**	**Oral Microbiota**	**Microbiota Collection Method**	**Association Between OD and AD**
**Sritana, N. and Phungpinii, A. 2024** [18]	Case-control study	*n* = 100 AD patients (*n* = 10); Mean age 66.9 y Patients with mild cognitive impairment (MCI) (*n* = 46); Mean age 68.5 y Healthy patients (*n* = 44); Mean age 64.73 y	*Cyanobacteria, Pseudomonadales, Fusobacteriota, Peptostreptococcaceae, Veillonella*	OMNIgene^®^ ORAL collection kit (DNA Genotek, Ottawa, ON, Canada) was used to collect saliva samples according to the manufacturer’s instructions.	YES
**Taati Moghadam M et al. 2022** [20]	Case-control study	*n* = 30 AD patients (*n* = 15) Healthy patients (*n* = 15)	*Porphyromonas gingivalis, Fusobacterium nucleatum, Prevotella intermedia, Aggregatibacter actinomycetemcomitans, Streptococcus mutans*	Oral bacterial microbiome composition was analyzed by quantitative real-time PCR (qPCR) using the 16S rDNA bacterial gene. Systemic inflammatory cytokine levels in both groups were assessed by ELISA.	YES
**Panzarella V et al. 2022** [21]	Case-control study	*n* = 60 AD subjects (*n* = 20) Subjects with amnestic mild cognitive impairment (aMCI) (*n* = 20) Controls (*n* = 20)	*Aggregatibacter actinomycetemcomitans (A.a), Fusobacterium nucleatum (F.* *n* *.), Porphyromonas gingivalis (P.g.), Prevotella intermedia (P.i.), Treponema denticola (T.d.), Tannerella forsythia (T.f.)*	Samples were collected from subgingival plaque bacterial load (using the Carpegen^®^ Perio Diagnostics kit with paper points on gingival crevicular fluid) for RT-PCR quantitative analysis of six periodontitis marker organisms.	YES
**Sansores-España LD et al. 2022** [22]	Case-control study	*n* = 30 AD patients (*n* = 10) Healthy patients (*n* = 20)	*Porphyromonas gingivalis*	Subgingival microbiota and GCF samples were collected from the deepest sites. Total DNA was isolated to quantify the 16S ribosomal subunit. Pro-inflammatory mediators and ApoE were quantified from gingival crevicular fluid (GCF).	YES
**Issilbayeya A et al. 2024** [23]	Case-control study	*n* = 135 AD patients (*n* = 64) Healthy patients (*n* = 71)	*Firmicutes, Bacteroidota, Haemophilus parainfluenzae, Prevotella melaninogenica, Prevotella histicola, Actinomyces oris, Limosilactobacillus, Lactobacillus, Lacticaseibacillus, Bacteroides, Catenibacterium, Parabacteroides, Eubacterium_eligens_group, Fusobacterium, Turicibacter, Anaerostipes genera*	Saliva was collected using a calibrated pipette from the floor of the mouth. Soft tissue samples were obtained from the dorsal tongue, hard palate, buccal mucosa, keratinized (attached) gingiva, palatine tonsils, and throat using a DNA/RNA shield collection tube with a swab. Supragingival and subgingival plaque were collected using a Gracey curette. DNA extraction was performed using the ZymoBIOMICS DNA miniprep kit.	YES
**Wu YF et al. 2021** [24]	Case-control study	*n* = 35 AD patients (*n* = 17) Healthy patients (*n* = 18)	*Firmicutes, Bacteroidetes, Fusobacteria, Fusobacterium, Cardiobacterium, Porphyromonas, Lactobacillus, Streptococcaceae, Actinomycetaceae, Veillonella*	Plaque was collected by a trained dentist using Gracey periodontal curettes. Genomic DNA was extracted using a bacterial genomic DNA kit.	YES
**Franciotti R et al. 2021** [27]	Case-control study	*n* = 78 Patients with neurodegenerative disease (*n* = 21) Patients with non-neurodegenerative disease (*n* = 28) Healthy patients (*n* = 29)	*Porphyromonas gingivalis*	Tongue biofilm was collected from each patient and control subject by the same dentist (P.P.) under identical conditions, 8 h after the last tooth brushing. The swab was obtained by brushing five times from the middle third of the tongue dorsum.	YES
**Qiu C et al. 2024** [25]	Cross-sectional study	AD patients (*n* = 32) Amnestic MCI patients (*n* = 32) Healthy patients (*n* = 32)	*Veillonella parvula, Lancefieldella parvula, Prevotella melaninogenica, Anaeroglobus geminatus, Streptococcus anginosus, Campylobacter gracilis, Dialister pneumosintes, [Eubacterium] yurii, Pseudoleptotrichia goodfellowii, Campylobacter rectus, Leptotrichia buccalis, Streptococcus sanguinis, Actinomyces massiliensis, Haemophilus parainfluenzae, Campylobacter concisus*	Subgingival plaque was obtained using Gracey curettes and placed in a sterile Eppendorf tube containing 0.5 mL of 1× phosphate-buffered solution (pH 7.2) and stored at −80 °C. Subgingival microbiota composition was determined by high-throughput sequencing of the 16S rRNA amplicon.	YES
**Holmer J et al., 2021.** [19]	Case-control study	*n* = 154 AD-diagnosed patients (50–80 y) (*n* = 52) Patients with mild cognitive impairment (*n* = 51) Patients with subjective cognitive decline (*n* = 51) Healthy patients (*n* = 76)	*Fusobacterium, Porphyromonas (P. gingivalis), Capnocytophaga, Treponema, Prevotella (P. intermedia), Campylobacter, Streptococcus, Slackia exigua, Lachnospiraceae, Actinomyces, Rothia*	Samples were collected using curettes; DNA extraction, PCR amplification, and sequencing of V3-V4 regions of the 16S rRNA gene were conducted by the DNA Sequencing and Genomics Laboratory, Institute of Biotechnology, University of Helsinki.	YES

## Data Availability

No new data were created or analyzed in this study.

## Abbreviations

AD	Alzheimer’s disease
BBB	Blood–brain barrier
PRISMA	Preferred Reporting Items for Systematic Reviews and Meta-Analysis
PG	Porphyromonas gingivalis
